# The Generation of a Comprehensive Spectral Library for the Analysis of the Guinea Pig Proteome by SWATH‐MS

**DOI:** 10.1002/pmic.201900156

**Published:** 2019-07-22

**Authors:** Pawel Palmowski, Rachael Watson, G. Nicholas Europe‐Finner, Magdalena Karolczak‐Bayatti, Andrew Porter, Achim Treumann, Michael J. Taggart

**Affiliations:** ^1^ Institute of Genetic Medicine Newcastle University Newcastle upon Tyne NE1 4EP Tyne and Wear UK; ^2^ School of Health Sciences Manchester University Manchester M13 9NT UK; ^3^ Newcastle University Protein and Proteomic Analysis Newcastle University Newcastle upon Tyne NE2 4HH Tyne and Wear UK

**Keywords:** guinea pig proteome, spectral library, SWATH‐MS

## Abstract

Advances in liquid chromatography‐mass spectrometry have facilitated the incorporation of proteomic studies to many biology experimental workflows. Data‐independent acquisition platforms, such as sequential window acquisition of all theoretical mass spectra (SWATH‐MS), offer several advantages for label‐free quantitative assessment of complex proteomes over data‐dependent acquisition (DDA) approaches. However, SWATH data interpretation requires spectral libraries as a detailed reference resource. The guinea pig (*Cavia porcellus*) is an excellent experimental model for translation to many aspects of human physiology and disease, yet there is limited experimental information regarding its proteome. To overcome this knowledge gap, a comprehensive spectral library of the guinea pig proteome is generated. Homogenates and tryptic digests are prepared from 16 tissues and subjected to >200 DDA runs. Analysis of >250 000 peptide‐spectrum matches resulted in a library of 73 594 peptides from 7666 proteins. Library validation is provided by i) analyzing externally derived SWATH files (https://doi.org/10.1016/j.jprot.2018.03.023) and comparing peptide intensity quantifications; ii) merging of externally derived data to the base library. This furnishes the research community with a comprehensive proteomic resource that will facilitate future molecular‐phenotypic studies using (re‐engaging) the guinea pig as an experimental model of relevance to human biology. The spectral library and raw data are freely accessible in the MassIVE repository (MSV000083199).

Advances in the speed, accuracy, and throughput of liquid chromatography mass spectrometry (LCMS) systems have brought proteomic workflows to the mainstream of biological experimentation. Data‐dependent acquisition MS modes (DDA), involving the most abundant eluted parent ions of an MS1 scan being selected for fragmentation in MS2 for peptide identification, have contributed considerably in this regard. However, the stochastic nature of parent ion selection can introduce variability to peptide identification outputs, hinder quantification between sample runs, and thus necessitate lengthy and costly procedures such as sample fractionation (to reduce input complexity) and injection replicates.^1^ The adoption of data‐independent acquisition (DIA) MS modes such as sequential window acquisition of all theoretical mass spectra (SWATH‐MS), whereby MS2 fragment ion spectra are collected for each parent ion observed, in a series of mass‐to‐charge isolation windows, has presented the opportunity to overcome these issues and obtain deep, label‐free proteomic coverage of complex samples in a timely manner without fractionation.^2^, ^3^, ^4^ Interpretation of SWATH spectra, however, requires reference to a spectral library of peptide sequence matching (including established *m*/*z* and LC retention time co‐ordinates), itself oft‐obtained from the outcomes of multiple DDA runs. Spectral libraries of notable depth are available for only a few species (or specialized cells/tissues)—including human,^5^ mouse,^6^, ^7^ a few microbiota,^8^, ^9^ drosophila and tomato,^10^ zebrafish,^11^, ^12^ and yeast^2^—that, at present, limits the breadth of uptake of SWATH.

The guinea pig is an excellent experimental model for many aspects of human physiology and pathophysiology—including maternal and fetal adaptations to pregnancy^13^, ^14^, ^15^ cardiac excitation‐contraction coupling,^16^ asthma and airway drug responsiveness,^17^ auditory somatosensory processes,^18^ type 2 diabetes,^19^ and vitamin C deficiency^20^—yet there is limited experimental information regarding the proteome available for this species. In an effort to overcome this obstacle, we therefore sought to generate a detailed spectral library of the guinea pig proteome.

The overall experimental workflow is displayed in **Figure** 1 and detailed in File S1, Supporting Information. Homogenates were prepared from 16 tissues (brain, colon, duodenum, adipose, kidney, large intestine, liver, lung, ovaries, pancreas, placenta, skeletal muscle, small intestine, stomach, heart, uterus) isolated from guinea pigs (fetal and adult) sacrificed according to the Animals (Scientific Procedures) Act 1986 under UK Home Office project license approval (PPL 60/4312). The study was approved by Newcastle University's ethics review process. Homogenates were trypsin digested and subjected to LC‐MS/MS DDA runs (Q‐Exactive or TripleTOF 6600) with differing chromatographic gradients and preprocessing steps (for details see Table S1, Supporting Information). No external calibrators were added to the tryptic digests (see File S1, Supporting Information for details of internal calibrators).

**Figure 1:**
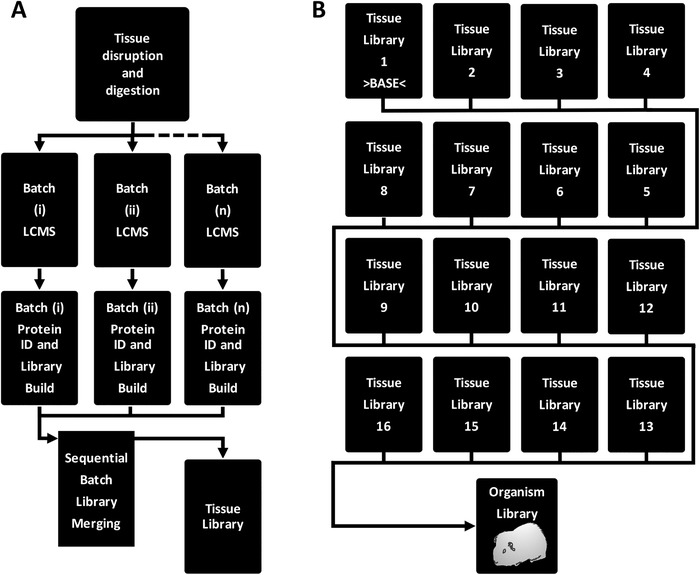
Schematic of the experimental workflow for spectral library generation. A) The steps to generate a tissue‐specific library. LCMS runs acquired for the same tissue on different occasions were classed as separate batches, searched using ProteinPilot and sequentially merged with SwathXtend to create a tissue‐specific library. B) Sequential merging of tissue libraries to create a multi‐tissue spectral library.

The acquired MS/MS data were searched against the Uniprot guinea pig proteome (version: January 2016) re‐annotated by combining the original annotation (if present) and annotation of homologous sequences from BLAST (version 2.2.30) searched against Swiss‐Prot mammalian sequences. Consistency of annotation (i.e., synonym elimination) was achieved by mapping to the HGNC database.^21^

The Q‐Exactive *.raw files were first converted to *.mgf format using MSConvert (ProteoWizard package). TripleTOF 6600 *.wiff files were searched directly, using Protein Pilot 5 (parameters: cysteine alkylation: iodoacetamide, digestion enzyme: trypsin, search effort: thorough, instrument: TripleTOF 6600/ Orbi MS, Orbi MS/MS, default settings). Joined searches were performed for LC‐MS runs of the same tissue, analyzed within the same experimental batch (same instrument and setup, acquired the same day) and showing the same peptide retention time profile (assed by visual inspection).

The individual search results were exported (using PeakView 2.1), in a spectral library format, as *.tsv files and sequentially merged into 16 tissue‐specific libraries using SwathXtend R package.^22^, ^23^ Prior to merging, the libraries were cleaned to only contain unmodified peptides identified with FDR < 0.01 with at least five corresponding fragment ions present. The confidence cutoff representative to FDR < 0.01 was applied individually to, each search result file. At each merge step, the retention times of the base and the add‐on library were aligned (**Figure** 2A) and the correctness (linearity, *R* ^2^ > 0.90) of the alignment was inspected. On occasions, the RT correlation was nonlinear, in order not to inadvertently lose high‐quality data from these situations, the gradient was manually divided into linear fragments, each of which was pre‐aligned with the base library, reassembled and then (if *R* ^2^ > 0.90, actual range 0.94–1) submitted to the SwathXtend merging algorithm (Figure 2B,C). Where the linear alignment was not possible (i.e., due to low correlation), the problematic portion of the add‐on library was removed. The assembly of the consensus spectral library was achieved analogically, by one‐by‐one joining of the individual tissue‐specific libraries (for detailed description see File S1, Supporting Information).

**Figure 2:**
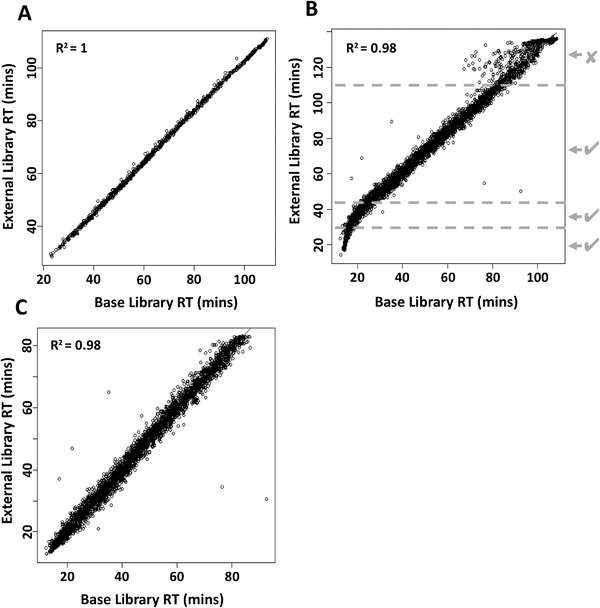
Concatenation of tissue‐specific spectral libraries. A) Liquid chromatography peptide retention time correlations between tissue‐specific libraries. (A) indicates excellent retention time correlation. B) indicates a situation where additional linearization was required. The external library was manually divided into four parts, three of which are the linear fragments of the plot and the 4th, noisy fragment, which was discarded. For each of the three linear fragments, the linear regression was calculated and the resulting parameters were used to adjust peptide retention times to match the base library. Subsequently, the fragments with corrected retention times were combined and used for library building. C) shows the outcome following normalization.

Analysis of >250 000 peptide‐spectrum matches resulted in the construction of a library of 73 594 peptides (unique to individual proteins) that corresponded to 7666 proteins. Seventy‐seven percent of proteins were identified with more than one peptide (**Figure** 3A). The contribution of tissue‐specific libraries to the total library varied roughly in accordance to the number of peptides, reflecting the biological properties, the number of biological replicates and repeat injections, and the level of fractionation that was carried out for different tissues (directed by the core research interests of our group) (Figure 3C). The overlap between tissue‐specific libraries is shown in Figure S1, Supporting Information, alongside normalized peptide counts plotted for proteins shared between the individual libraries, providing an indication of how similar/dissimilar different tissues are in terms of protein composition. Peptide retention time correlations and the corresponding correlation residuals plots are provided as Figure S2, Supporting Information.

**Figure 3:**
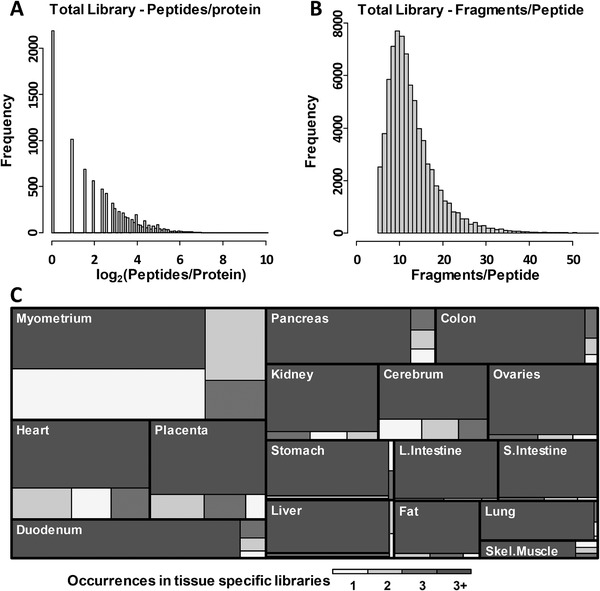
Summaries of library composition. Histograms indicating A) the number of peptides per protein and B) the frequency distributions of fragment ions per peptide. C) Tissue‐specific library contributions to the total library. Different colors indicate what proportion of the library is shared between multiple tissues (from 1 to 3+, 1 being unique to one tissue only and 3+ being found in more than three tissues).

To demonstrate i) the applicability of our spectral library to be used for analysis of any guinea‐pig‐derived SWATH data and ii) the potential for incorporation of other available resources (other libraries or re‐analyzed DDA data), we accessed externally acquired guinea pig retinal SWATH data published by Shan et al., 24, 25 and the corresponding retinal spectral library. Three biological replicate SWATH files (Day 21, left eye) were analyzed in parallel using i) our guinea pig spectral library, having first assured satisfactory RT correlation with the SWATH runs (Figure S3A,B, Supporting Information) and ii) the retinal tissue spectral library of Shan et al.^24^, ^25^ Assessment of the peptide quantification reproducibility showed very good consistency between both replicate samples and libraries, that is, the peak volume correlation for peptides shared between both libraries was *R* ^2^ > 0.94 (Figure S3C–E, Supporting Information). This demonstrated the usefulness of our library for other users and their SWATH analysis. It also shows that in absence of a dedicated tissue‐specific library, a broad external library may already allow reliable protein quantification with good proteome coverage.

Moreover, a comparison of the multi‐tissue guinea pig spectral library and the retinal tissue‐specific library showed considerable overlap in peptides (73%, Figure S4A, Supporting Information) and proteins (91%, Figure S4B, Supporting Information). Nonetheless, it was of interest to attempt to merge the libraries and facilitate accessing all available information in one search space. Therefore, utilizing the same procedure that was used in the process of our library building described above, retinal DDA files were successfully merged to our guinea pig library (for more details see Figure S4A–D and File S1, Supporting Information). This merged spectral library increased the number of peptides by 3907 and proteins by 270. Analyzing the three retinal SWATH files with this library now resulted in quantification of 445 retinal tissue proteins (Figure S4E, Supporting Information) not reported by Shan et al.^24^, ^25^

In summary, we have generated a detailed spectral library of the guinea pig proteome for interrogation by SWATH‐MS that greatly increases the validated guinea pig proteome information. We have demonstrated the usefulness of our library for other users and their SWATH analysis, and how external data (libraries, search results) can also be incorporated to our base library. Also of note, in the absence of users having a dedicated tissue‐specific library (e.g., due to cost restraints), our library may be used for reliable protein quantification with a good proteome coverage. We provide it as a tab delaminated text file, formatted to be compatible with PeakView and also easily converted for use by Skyline, OpenSWATH, or the latest versions of Spectronaut. The freely accessible library will thus furnish the research community with a resource to i) iteratively add new data to; ii) explore future proteome‐phenotypic studies using (re‐engaging) the guinea pig as an experimental model; and iii) assess, if desired, cross‐species proteomic responses to consistent physiological and pathophysiological experimental challenges.

## Conflict of Interest

The authors declare no conflict of interest.

## Supporting information

Supporting Information
