# Supplementary material for: The Generation of a Comprehensive Spectral Library for the Analysis of the Guinea Pig Proteome by SWATH‐MS
Source: Proteomics. 2019 Jul 22;19(15):1900156. doi: 10.1002/pmic.201900156 (PMC6771470; doi:10.1002/pmic.201900156)
Supplement: Supplementary file 1 — Supporting Information [file PMIC-19-na-s001.zip › Supplementary Figure legends.docx]

**Supplementary Figure 1. Tissue-specific library overlaps in protein and peptides.**

The overlap of identified proteins between individual tissue-specific libraries (above diagonal) contributing to the total library. The corresponding comparison of normalized peptide counts per protein shared between tissue specific libraries are displayed below the diagonal (peptide counts displayed as log2 values). Tissues analysed in this study with corresponding number of LC-MS runs included in the spectral library are indicated on the diagonal.

**Supplementary Figure 2. Retention time correlation\ residual plot.**

The peptide retention time correlation between individual tissue specific libraries (above diagonal) and corresponding residual plots (below diagonal)

**Supplementary Figure 3. Externally-derived SWATH data file analysis.** A, liquid chromatography retention time (RT) correlation of the multi-tissue spectral library (y-axis) to one retinal SWATH data file (x-axis). The RT correlation was not satisfactory at later retention times. B, after alignment correction the RT correlation was satisfactory R^2^ = 0.97. The aligned library was then used to analyse the retinal SWATH data. C-D, peptide intensity correlation of two SWATH retinal data files analysed with the multi-tissue library (C) or the retinal tissue-specific library (D). E, peptide intensity correlations from three retinal SWATH data files analysed against the multi-tissue library (y-axis) or the retinal tissue-specific library (x-axis). Red and green points (on the axes) represent intensities of peptides quantified with only one of the libraries.

**Supplementary Figure 4. Merging of externally-derived retinal data to the guinea pig proteome spectral library.** There was substantial overlap in peptides (A) and proteins (B) identified in the retinal tissue-specific library and that of the multi-tissue library. To coalesce non-overlapping information, merging of the retinal tissue-specific library to the multi-tissue library was attempted. Initial RT correlation between the libraries (C) required realignment following which (D) the correlation was satisfactory R^2^ = 0.99. E, protein identifications from three retinal SWATH files when analysed with the retinal tissue-specific library, the multi-tissue library or the merged retinal-multi-tissue libraries.

**Supplementary Figure 5. Spectral libraries – peptide SWATH comparison.**

(A) Identity of peptides quantified in the analysed SWATH files using the external (multi-tissue) library, dedicated (Retinal, sample specific) library and a combination of both. There is a population of peptides uniquely identified using only one library. (B) Peptide intensity (peak area) in peptides identified by one, two or all the above libraries.

**Supplementary Table 1. Summary experimental procedures and data analysis.**

The details of individual sample processing procedures, LC-MS methods and instrumentation used, together with MS-MS searching information (searching batch composition)
