# Supplementary material for: The Generation of a Comprehensive Spectral Library for the Analysis of the Guinea Pig Proteome by SWATH‐MS
Source: Proteomics. 2019 Jul 22;19(15):1900156. doi: 10.1002/pmic.201900156 (PMC6771470; doi:10.1002/pmic.201900156)
Supplement: Supplementary file 1 — Supporting Information [file PMIC-19-na-s001.zip › Supplementary File 1 - extended Methods.docx]

This document describes the methodological details of (1) Spectral library assembly and (2) Guinea pig library validation with analyses of external data files.

**(1) Spectral library assembly.**

Guinea pig samples prepared using 16 tissues (brain, colon, duodenum, adipose, kidney, large intestine, liver, lung, ovaries, pancreas, placenta, skeletal muscle, small intestine, stomach, heart, uterus) were analysed in >200 LC-MS/MS DDA runs using two instrument platforms (Q-Exactive or TripleTOF 6600) with differing chromatographic gradients and pre-processing steps, by different laboratories. The acquired data was subjected to multiple MS/MS ion searches. The results of these searches were used to assemble 16 individual tissue-specific spectral libraries and, subsequently, 1 combined Guinea pig multi-tissue library, to be used for SWATH data analysis. The procedure to accomplish these tasks was as follows:

1. **Files grouping:**

The LC-MS result files were grouped into “batches”. Each batch would only contain files acquired using the exact same instrument setup on the same day. The files within each batch would be then assessed (by visual inspection) for consistent peptide retention time (RT). In order to do this, files were individually subjected to a preliminary database search: the RTs were plotted against each other and compared. Outlying files were excluded from a batch.

1. **MS/MS Ions Search:**

- The Q-Exactive *.raw files were first converted to *.mgf format using MSConvert (ProteoWizard package).
- TripleTOF 6600 *.wiff files were searched directly.
- For database searching we used Protein Pilot 5 (parameters: cysteine alkylation: iodoacetamide, digestion enzyme: trypsin, search effort: thorough, instrument: TripleTOF 6600/ Orbi MS, Orbi MS/MS, default settings).
- The individual search results (*.group files) were converted (using PeakView 2.1) to a spectral library format (*.tsv files)

1. **Tissue-specific libraries assembly**

16 tissue specific spectral libraries were built. Each spectral library consisted of multiple search result files (“batch specific libraries”) merged together sequentially (the number of “batch specific libraries would vary between tissues) using SwathXtend R package [22-23]. To do this required RT alignment as follows:

- - **RT alignment:**
    1. Two libraries (one being the base library and the other one being add-on library) were loaded into R.
    2. Confidence cut-off was extracted from the corresponding ProteinPilot search summaries in .xlsx format that were equivalent to identification FDR<0.01.
    3. The libraries were cleaned to only contain unmodified peptides identified with FDR<0.01 with at least 5 corresponding fragment ions present.
    4. The SwathXtend package was used to check how well peptide RT’s matched between libraries. The program would plot RTs of all the peptides shared by the libraries being merged and calculate a R^2^ value.
    5. If the RT correlation was linear, with R^2^>0.9, no further processing was required.
    6. On occasions, the RT correlation wasn’t satisfactory, or contained a visibly non-linear portion(s). In such instances, in order to assure RT consistency, the add-on library gradient was pre-aligned with the base library before merging as follows:
       - 1. The add-on library was divided into linear fragments. The initial number of fragments was based on visual inspection of the RT plot
         2. Each of the fragments was inspected again to check for any additional segmentation and further fragmented, if needed.
         3. Linear regression was calculated for each selected fragment. Any neighbouring segments, for which the slope difference was >0.15 were considered separately for the purposes of RT alignment.
         4. The exact boundaries between neighbouring linear fragments were determined first using an intersection of the corresponding linear regression lines and then adjusted within a temporal range of 1-2 minutes range, to form a continues distribution when reassembled (see steps below).
         5. The regression line parameters were used to adjust peptide RTs in the add-on library.
         6. On rare occasions, where the linear alignment wasn’t possible/satisfactory (i.e. bad correlation, typically towards the end of the gradient), the problematic portion of the add-on library was removed before reassembling.
         7. The pre-aligned linear fragments were reassembled.
  - **Merging batch-specific libraries to make a tissue-specific library.**
    1. When the correlation was linear and R^2^>0.90 (actual range 0.94-1) the two libraries intended to be merged could be submitted to the SwathXtend merging algorithm.
    2. The resulting combined library was then used as a base in subsequent rounds of merging cycles until all the “batch specific” libraries were combined into one tissue-specific library.
    3. The resulting tissue-specific library was saved in PeakView format as *.txt file.

The whole process of assembling the tissue-specific libraries was documented in the form of R scripts provided as supplementary files. These are: individual_tissue_specific.R, heart library.R, myo_library.R. The Myomerium (myo) and heart libraries are dealt with in separate scripts due to their large size and number of merging cycles involved.

1. **Guinea pig multi-tissue library assembly**

The 16 tissue-specific libraries were combined into one Guinea pig multi-tissue library following a similar procedure as described above:

- - 1. Two libraries (one being a base library and the other one being add-on library) were loaded into R.
    2. There was no cleaning required at this stage, as these libraries were cleaned via the above procedure (section 3).
    3. The SwathXtend package was used to check how well peptide RT’s match between libraries.
    4. RT pre-alignment was done, when needed, as described for tissue-specific library assembly (see section 3).
    5. When the correlation was linear and R^2^>0.90 (actual range 0.94-1) the two libraries intended to be merged could be submitted to the SwathXtend merging algorithm.
    6. The resulting library was saved in PeakView format as *.txt file.

**(2) Guinea Pig Library validation**

To validate the guinea pig spectral library we utilised publicly available external data, published and kindly supplied by Shan et al., [references 23-24]. These authors had built a retinal tissue-specific library from guinea pigs, consisting of 3138 proteins (22,871 peptides) which they used to analyse the SWATH data acquired from the same retinal tissue sample set. As retina was not among the 16 tissues furnishing our multi-tissue guinea pig spectral library, this was an appropriate dataset to access. The retinal data was acquired using a TripleTOF 6600 mass spectrometer. Samples were loaded at a flow rate of 3 μl/min and eluted from the analytical column at a flow rate of 300 nl/min with a linear gradient of 6.8% to 35.6% acetonitrile in 120 min [23-24].

Two validation tasks were attempted: the use of the guinea pig library to analyse retinal SWATH data and the outcome of attempting to merge the guinea pig library with the retinal tissue-specific spectral library.

**Retinal SWATH data analysis.**

The procedure for retinal SWATH data analysis with our guinea pig library was as follows:

**(a) Retention time pre-alignment:**

In order for any external SWATH data to be analysed with the multi-tissue guinea pig library a best possible correlation needed to be assured between the retinal SWATH runs and the spectral library. The process of aligning these was as follows:

- - 1. **Swath file conversion:** one of the retina SWATH files was converted to *.mgf format using DIAUmpire (Chih-Chiang Tsou et al., Nat Methods. 2015;12:258-64).
    2. **MS/MS Ions Search:** the resulting *.mgf file was searched against the Guinea pig protein database using ProteinPilot 5 (parameters used: cysteine alkylation: iodoacetamide, digestion enzyme: trypsin, search effort: thorough, instrument: TripleTOF 6600, default settings). The resulting ProteinPilot *.group file was subsequently exported to a spectral library format (*.tsv) in PeakView v2.1.
    3. **Retention time correlation check:** The converted retinal SWATH file search result (in spectral library format) was loaded into R and the SwathXtend package was used to check how well peptide RT’s matched to our guinea pig multi-tissue library. As shown in the Supplementary Figure 3A, the RT correlation was not satisfactory (particularly at later retention times), which is a consequence of differences in the chromatographic gradients used in our study and that of [23-24].
    4. **Library RT alignment:** to better match the guinea pig library to peptide elution profiles in the to-be analysed retinal SWATH file, we applied the same procedure that we already described above for merging batch- or tissue-specific libraries using the SwathXtend R package. The whole process was documented in the “total_aligned_to_retina.R” script. This resulted in a much improved RT correlation (supplementary Figure 3B). The aligned spectral library was saved and used for subsequent analysis of the retinal SWATH files.

**(b) SWATH file analyses:**

Three biological replicate SWATH retinal files (Day 21, left eye) were analysed in parallel using (i) our realigned guinea pig spectral library and (ii) the tissue-specific retinal library of [23-24] (with PeakView 2.1, SWATH micro app, all peptides used for quantification, XIC window = 10, max. mass error=10ppm). For RT calibration a set of parent ions was chosen individually for each library, which were high intensity and with symmetrical, sharp peaks in all analysed SWATH files.

The resulting outputs were then exported to *.txt file format and further processed in Perseus software platform (Tyanova et al., (2016) *Nature Methods* **13**, 731).

**(c)** **Results:**

The two analyses combined resulted in the identification/quantification of 11975 peptides (2480 proteins). The quantification reproducibility for the two libraries was initially assessed by comparing the calculated peptide peak volumes (transformed to log scale) obtained between two biological replicates. As shown in Supplementary Figure 3C-D, these SWATH data showed excellent consistency when analysed with each library. Subsequently, using data averaged from the three biological replicate SWATH runs enabled a direct comparison of (common) peptide intensities obtained using each library to be made. This produced a correlation R^2^ of 0.94 (Supplementary Figure 3E).

**Incorporation of the external tissue-specific retinal library**

When directly comparing (the same filtering was applied to both libraries), our multi-tissue and the retinal spectral libraries there was a substantial overlap (see Supplementary Figure 4A-B). Nonetheless 3907 peptides and 270 proteins of the retinal tissue-specific library were not identified in the multi-tissue guinea pig library. Therefore, the potential to incorporate new externally-acquired data into our library was investigated using the retinal spectral library data files of [23-24]. We utilised the same procedure as that already described above for the spectral library building process.

- The multi-tissue guinea pig library, and the tissue-specific retina library, were loaded in to R in a *.tsv format.
- The SwathXtend package was used to check for retention time correlation between the base library (the multi-tissue guinea pig library) and the incoming library (the tissue-specific retinal library).
- For RT correction, the gradient was divided into 3 linear fragments, each of which was pre-aligned and reassembled (Supplementary Figure 4C-D; for detailed documentation see “total_and_retina_combined.R” script).

The two libraries were merged (using SwathXtend), expanding the base library with 270 proteins and 3907 additional retina specific peptides. For validation purposes the retinal SWATH files were then reanalysed using this expanded library. This resulted in improved proteome coverage (see Supplementary Figure 4E).
