# Supplementary figures and images for: The Generation of a Comprehensive Spectral Library for the Analysis of the Guinea Pig Proteome by SWATH‐MS
Source: Proteomics. 2019 Jul 22;19(15):1900156. doi: 10.1002/pmic.201900156 (PMC6771470; doi:10.1002/pmic.201900156)

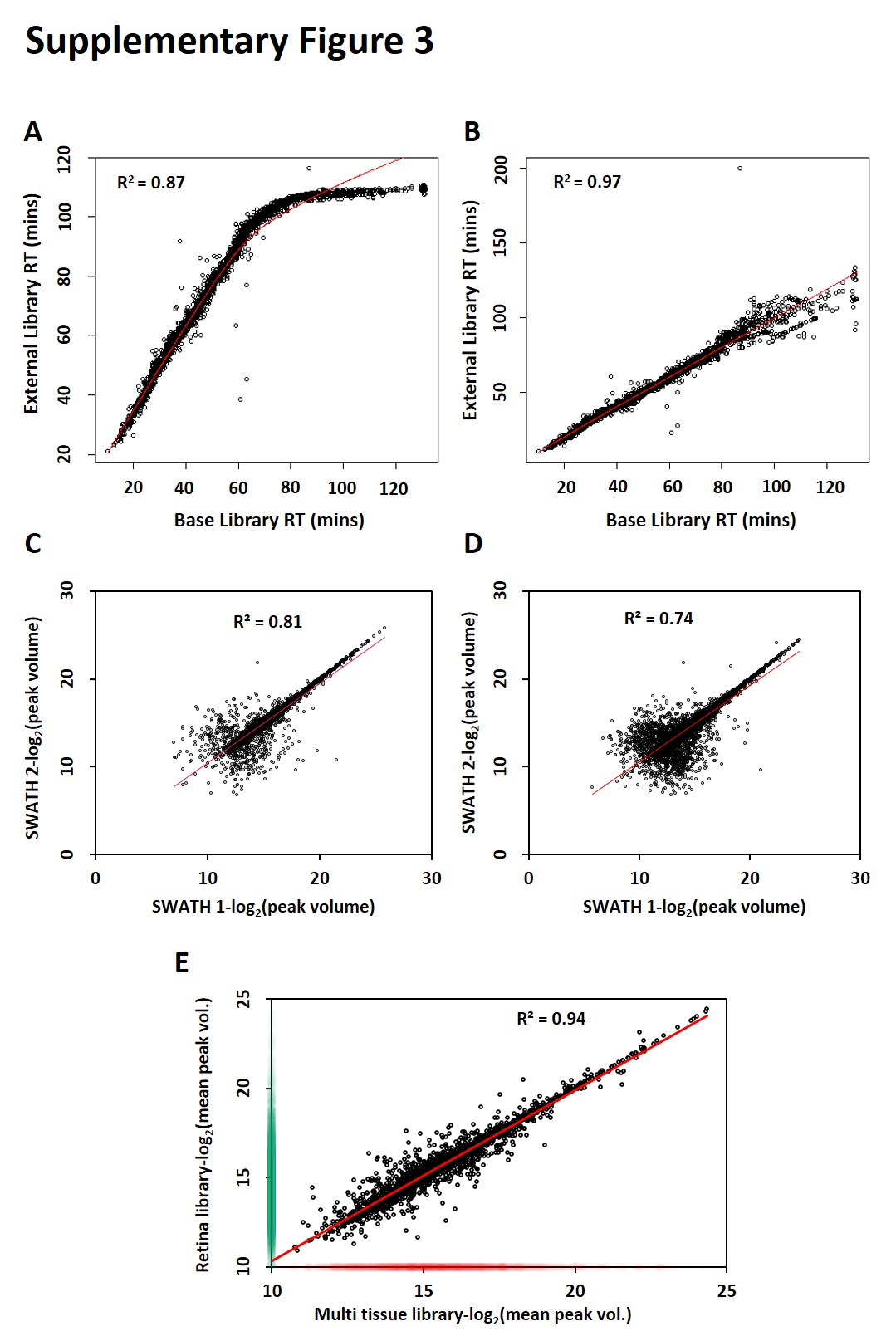

Supplement: Supplementary file 1 — Supporting Information [file PMIC-19-na-s001.zip › Supplementary figure 3.jpg]

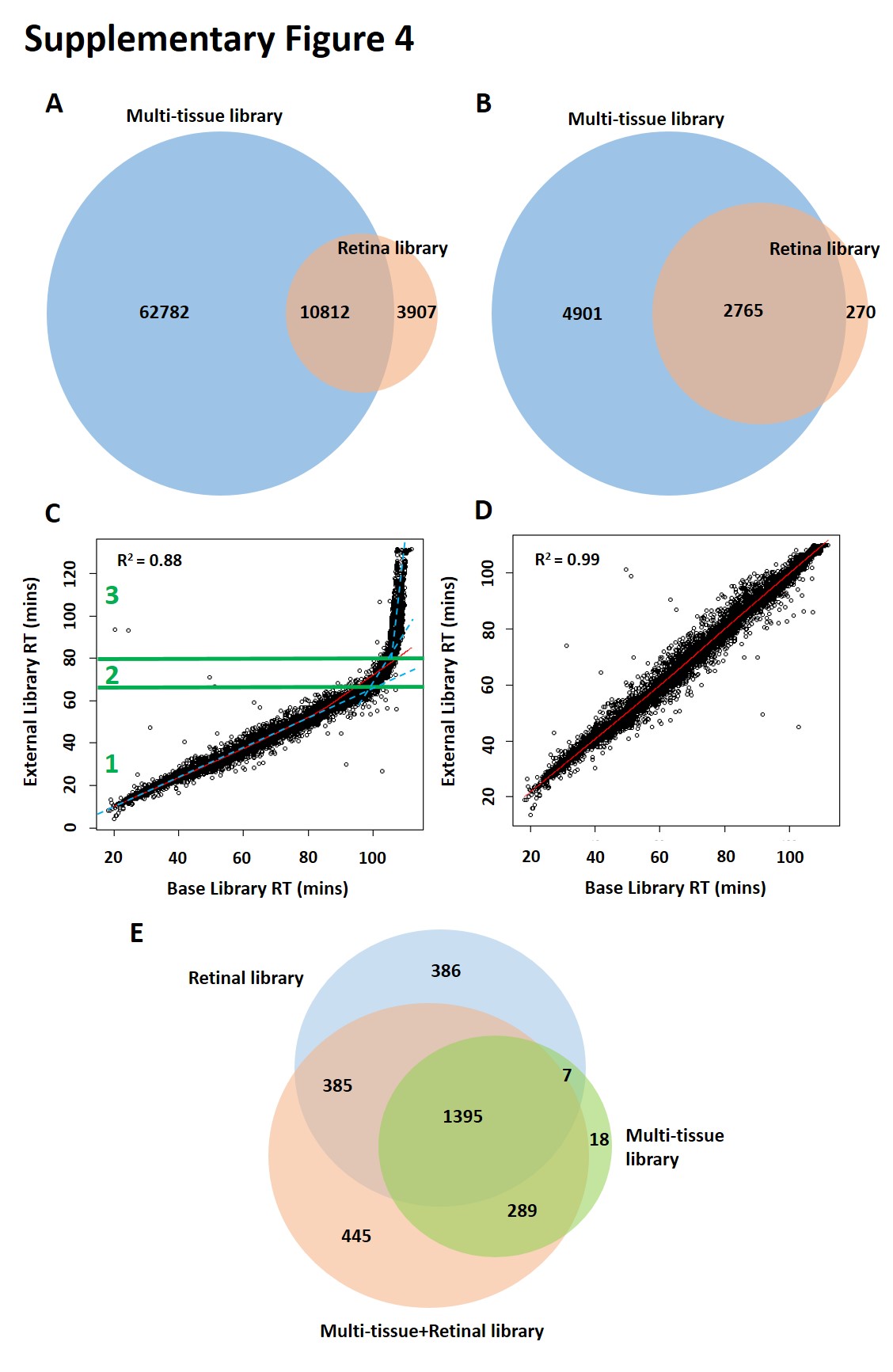

Supplement: Supplementary file 1 — Supporting Information [file PMIC-19-na-s001.zip › Supplementary figure 4.jpg]

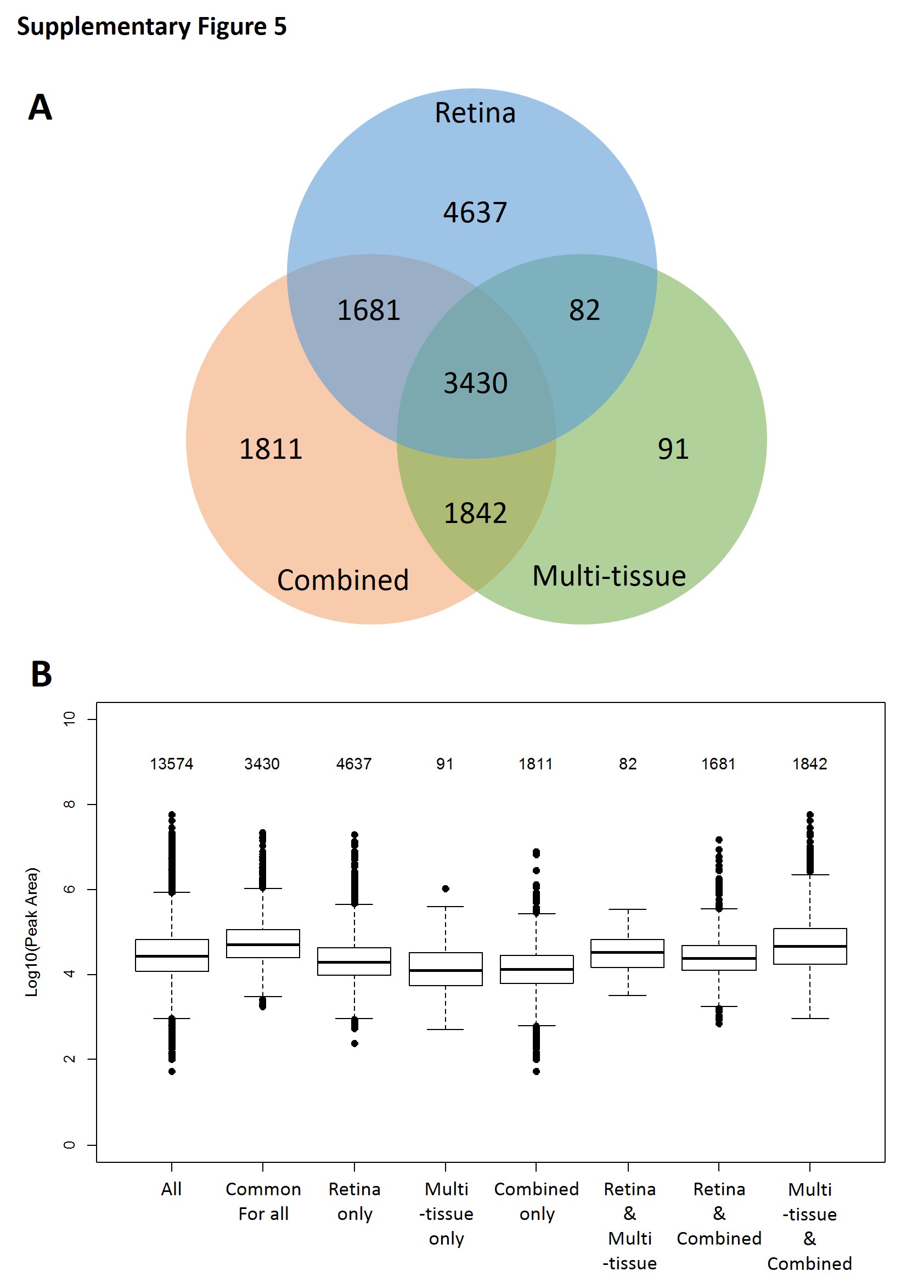

Supplement: Supplementary file 1 — Supporting Information [file PMIC-19-na-s001.zip › Supplementary figure 5.jpg]
